# Endocrine Disruptor Potential of Short- and Long-Chain Perfluoroalkyl Substances (PFASs)—A Synthesis of Current Knowledge with Proposal of Molecular Mechanism

**DOI:** 10.3390/ijms22042148

**Published:** 2021-02-21

**Authors:** Katarzyna Mokra

**Affiliations:** Department of Environmental Pollution Biophysics, Faculty of Biology and Environmental Protection, University of Lodz, Pomorska 141/143 St., 90-236 Lodz, Poland; katarzyna.mokra@biol.uni.lodz.pl

**Keywords:** PFOS, PFOA, PFHxS, perfluoroalkyl substances, short-chain PFASs, endocrine disruptor, prenatal exposure

## Abstract

Endocrine disruptors are a group of chemical compounds that, even in low concentrations, cause a hormonal imbalance in the body, contributing to the development of various harmful health disorders. Many industry compounds, due to their important commercial value and numerous applications, are produced on a global scale, while the mechanism of their endocrine action has not been fully understood. In recent years, per- and polyfluoroalkyl substances (PFASs) have gained the interest of major international health organizations, and thus more and more studies have been aimed to explain the toxicity of these compounds. PFASs were firstly synthesized in the 1950s and broadly used in the industry in the production of firefighting agents, cosmetics and herbicides. The numerous industrial applications of PFASs, combined with the exceptionally long half-life of these substances in the human body and extreme environmental persistence, result in a common and chronic exposure of the general population to their action. Available data have suggested that human exposure to PFASs can occur during different stages of development and may cause short- or/and long-term health effects. This paper synthetizes the current literature reports on the presence, bioaccumulation and, particularly, endocrine toxicity of selected long- and short-chain PFASs, with a special emphasis on the mechanisms underlying their endocrine actions.

## Highlights

1. Short-chain PFASs, similar to long-chain PFASs, are widely distributed in biotic and abiotic components of the environment.
2. Health effects caused by PFASs exposure are related to endocrine disruption and varied according to gender and age of development.
3. Obtained data showed that both long- and short-chain PFASs exhibit potential impacts on steroid hormone precursor (DHEA, aldosterone) level.
4. In many cases short-chain PFASs exhibit similar or even higher endocrine disrupting potential than long-chain PFASs (especially PFHxS).
5. In vivo and in vitro studies have reported that PFASs can bind to nuclear receptors, such as estrogen receptors (ERs), androgen receptor (ARs) and thyroid hormone receptor (TRs); therefore, they are can alter steroidogenesis.

## 1. Introduction

Endocrine disrupting chemicals (EDCs) are largely widespread compounds in the environment and human surroundings, exhibit hormone-like properties even in low-doses and induce adverse health effects associated with hormonal system deregulation [1]. The mechanisms of EDCs action are complex and can be mediated both by the genomic (involving intracellular, nuclear and cytoplasmic receptors) and by the pathway via membrane receptors and secondary signaling transductors [2]. Despite of a large body of scientific evidence, the mechanisms of action of per- and polyfluoroalkyl substances (PFASs) are still unclear.

### 1.1. PFASs Chemical Structure and Classification

PFASs are an extensive group of chemicals of anthropogenic origin. There are currently 4730 compounds classified as PFAS-related CAS numbers [3]. They are divided into several subgroups of compounds demonstrating various properties. PFASs are chemical entities in which all or a majority of hydrogen atoms in the carbon chain are substituted by fluorine atoms (Table 1). There are perfluoroalkyl acids (PFAAs), including perfluoroalkyl carboxylic acids (PFCAs) with compounds possessing seven or more perfluorinated carbon atoms (C_n_F_2n+1_COOH, e.g., perfluorooctanoic acid (PFOA), perfluoroalkane sulfonic acids (PFSAs) with compounds possessing six or more perfluorinated carbon atoms (CnF_2n+1_SO_3_H, e.g., perfluorooctanesulfonic acid, PFOS) and perfluoroalkane sulfonamide acids (e.g., perfluorooctane sulfonamide, PFOSA) [3]. PFASs are a particularly intensively studied subgroup, because of their significant presence in the environment. PFASs were first produced in 1950s, and they are still broadly used in the industry, for example, in the production of firefighting agents, solvents, cosmetics, textile protecting agents (Gore-Tex textile), herbicides, floor polishes, paints and kitchenware surface covers (Teflon) [4,5], as well as in the production of food packaging, including fast-food packages [6]. Considering their particular physical and chemical properties (such as the ability to lower the surface tension during metal plating or ability to alter the electrical potential at the metal component surface and prevent its electrochemical oxidation), PFASs are also used in the motor, aviation, construction and electronic industries [6].

### 1.2. PFASs Laws and Regulations

PFASs are characterized by a high persistence; the half-life of some of them in the environment is as long as 94 years, and biochemical half-life of PFOA, PFOS and perfluorohexane sulfonic acid (PFHxS) in human blood is 3.8, 5.4 and 8.5 years, respectively [7,8]. PFASs are present in the environment in the form of completely dissociated anions [9] that easily accumulate in surface waters [10]. In 2006, the Scientific Committee of Health and Environmental Risks (SCHER) classified PFOS as highly bio-resistant substances and classified them as persistent organic pollutants (POPs). In 2006, the European Union adopted the Directive (2006/122/EC) stating that, after the year 2008, the maximum PFOS content in a semi-product could be up to 0.005% of its total weight. In 2009, PFOSs were included in the list B of the Stockholm Convention, thus limiting (not banning) their use in the countries that had not previously regulated the industrial use of these chemicals.

Pursuant to the Stockholm Convention, the use of two main representatives of PFASs–PFOS and PFOA—should be limited by the end of 2020, and the US Environmental Protection Agency (US EPA) developed a plan assuming withdrawal of PFOA and its derivatives by eight main manufacturers of fluoropolymers (Arkema, Asahi, Daikin, BASF, Clariant, DuPont, 3M and Solvay Solexis), by the year 2015, in the US territory. Regulations of industrial use of PFOS were also introduced by Japan, Eastern Europe, Canada and Australia [11,12]. China, where annual production of PFOS has increased from 30 tons in 2002 to 247 tons in 2006 [13], decided to reduce their production, considering international legislation. The Chinese plan for the environment protection, developed in 2016, assumed withdrawal of PFOS and their derivatives from production by the year 2020 [14].

Despite of the above limitations, China is still one of the biggest producers and importers of PFASs, and, at the same time, it is the biggest user of products based on per- and polyfluorinated compounds [15,16,17]. Moreover, despite the reduction or even withdrawal of long-chain PFASs from production by the largest chemical producers in the world, some manufacturing plants in Asia continue to use PFASs (including PFOS) and their precursors for production and processing [18,19].

### 1.3. Long-Chain PFASs Alternatives

Long-chain PFASs (having a chain composed of seven or more carbon atoms) are being replaced with other products, such as other fluorine-containing compounds, mostly short-chain PFASs (possessing the chain composed of six or less carbon atoms) and by compounds without fluorine substituent. Despite the efforts to find the replacements deprived of fluoride atoms, including those based on dendrimers, nanomaterials, stearin or paraffin [20], none of them has shown such strong amphiphilic properties, which are offered by perfluorinated compounds. Then, the water- and oil-resistant effects of alternatives are incomparably weaker when compared with PFASs. Among replacements of long-chain PFASs used currently by the industry, there are short-chain PFASs (C4–C6), including perfluorohexane acid (PFHxA) or perfluorobutane sulfonic acid (PFBS). Some reports have indicated a higher environmental mobility of short-chain PFASs, compared to their long-chain equivalents [21]. It has been revealed that biochemical PFHxS half-life in the human blood (young females) is longer than that of PFOS [22,23]. In 2003, the 3M consortium replaced PFOS with PFBS, thus completing the declaration of withdrawal of long-chain PFASs and, at the same time, accounting for increased environmental presence of alternative PFASs. Moreover, one of technologies aimed at replacement of six- and eight-carbon PFASs by introduction of the PFBS precursor-perfluorobutane-sufonyl fluoride [24].

The recent use of PFBS is reflected by increased content of this substance in some washing agents, e.g., carpet shampoos [25]. The study from 2012 [26], in which selected PFASs content in blood of pregnant women (Sweden) was measured in 1996–2010 confirmed the observations of other researchers regarding reduced level of PFOS and PFOA in blood of the general population with simultaneous significant increase of the short-chain PFASs content, including PFBS and PFHxS. Moreover, the abovementioned analysis indicated that the significant increase of PFBS and PFHxS levels in women’s blood (by 11% and 8.1%, respectively) occurred at the time of withdrawal and limited industrial use of PFOA and PFOS.

### 1.4. Health Effects Caused by PFASs

The exposure to PFASs is known to cause liver toxicity, reproductive disorders, neurotoxicity and immunotoxicity (Table 2). Harmful health effects observed as a result of PFASs exposure could be highly associated with disturbance of hormone homeostasis. It has been reported that PFASs could interfere with molecular components of the endocrine system and modulate synthesis or secretions of selected hormones [27,28,29]. PFOA and PFOS act as endocrine disruptors mainly via effect on distribution of sex hormones, through mechanisms related to estrogen receptor activation and transcription of selected genes [29,30,31]. An in vivo and in vitro study conducted on animals have shown negative impact of two short-chain PFASs, i.e., PFBS and PFHxS on reproduction through the hypothalamus–pituitary–gonad axis [32], mainly due to deregulation of thyroid function [33,34,35,36]. Epidemiologic evidence of endocrine-disrupting activity of short-chain PFASs is limited and, similar to study on long-chain PFASs, in many cases inconsistent. As a result, none of PFASs has been categorized as EDCs by any legislative bodies up to these days. The main reason for considering these compounds to be endocrine-toxic was based on consistent reports, showing thyroid hormone level alterations and high risk of hypothalamic–pituitary–gonadal axis in animals exposed to PFOS [37,38,39,40].

### 1.5. The Aim of the Review

This study focuses on endocrine toxicity of two representatives of long-chain PFASs, i.e., PFOA and PFOS, and the representatives of short-chain compounds (PFBS, PFHxS, PFBA, PFHxA, PFNA and PFDA), which are the most commonly detected in serum and urine of humans. Considering the fact that PFASs have been using for many years up to now, their presence in the environment is common. Therefore, in light of some reports indicating a comparable toxicity of long-chain PFASs and their short-chain equivalents, an overview of the most current literature findings about endocrine impact of these xenobiotics is advisable. Replacing harmful chemicals with the compounds belonging to the same chemical group always raises some doubts, often legitimate. An example may be a replacement of bisphenol A (BPA) with bisphenol AF (BPAF), which has been shown to be more toxic for human red blood cells (RBCs) and peripheral blood mononuclear cells (PBMCs) [60,61,62,63].

First sections of the review introduce chemical structure, sources of the exposure and transformation of PFASs in living organisms, as well as their occurrence in the environment. The goal of the last section, “Endocrine Disruption Caused by PFASs”, was to assess endocrine toxicity of selected PFASs and propose molecular mechanism of action of these substances.

## 2. Physicochemical Properties

**Figure 1:**
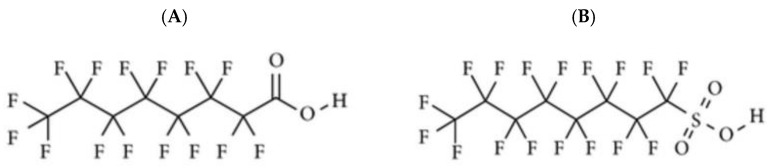
Chemical structures of perfluorooctanoic acid (PFOA) (**A**) and perfluorooctane sulfonic acid (PFOS) (**B**).

The basis of PFOA, PFOS and short-chain PFASs is a hydrophobic, n-carbon chain, in which each carbon atom is substituted with fluorine atoms (perfluoroalkyl chain) (Figure 1). A functional group demonstrating hydrophilic properties is attached to the chain [64]. Contrary to other persistent organic pollutants (POPs), PFASs are amphiphilic. Amphiphilicity means that a compound contains both strongly hydrophilic (charged end of the perfluorocarbon chain) and strongly oleo- and hydrophobic regions [65,66]. In practice, this means that during contact of, PFASs-coated material with water or oil, the interaction between CF_2_ molecules in PFASs will be extremely weak compared to the interactions between the water or oil molecules themselves. As a result, this leads to externalization (or rather pushing out) of hydrocarbon chains of the water/fat/oil molecule and to formation of spherical structures (droplets) of these substances on the surface of coated material. PFASs exist both in neutral and anionic forms, while the anionic form makes those compounds more easily soluble. Solubility of PFOA is 9.5 g/L (at room temperature, 25 °C). It is therefore higher than solubility of polybrominate diphenyl eters (PBDEs) regarded as one of the easiest water-soluble POPs [67]. In the environment PFOA and PFOS are present mostly in the anionic form, resulting on easy accumulation of those compounds in both biotic and abiotic water-containing components. The bioavailability of xenobiotics is dependent on several chemical parameters, including its pKa values. Amount of compound that exist in unionized and in ionized form is a function of pKa of compound and pH of solvent. The pKa value for highly bioavailable PFOA ranges from 0 to 3.8 [68], although bioavailability of PFASs is the most commonly discussed not in relation to the pKa value itself, but in relation to kinetic behavior [69]. This is in more details discussed in the section regarding PFASs accumulation in living organisms. PFASs are resistant to photolysis and to reactions with acids, alkalis and reducing agents [70,71,72,73,74]. Chemical and physical properties of PFOS, PFOA and PFBS are presented in Table 3 [70,71,72].

## 3. Sources of Exposure and Transformation in Living Organisms

### 3.1. Sources of Exposure

Studies indicated that 95% of US citizens had PFASs in their blood serum [75]. Food and drinking water are the main sources of the exposure to PFASs. Data published in 2018 indicated that tap water and bottled water collected in Canada, USA, Chile, Burkina Faso, Ivory Coast, France, Japan, Norway and Mexico were contaminated with PFBS at the concentrations from 0.2 to 1.6 ng/mL [76]. The study carried out in 79 cities of 31 administrative regions of China demonstrated that mean PFASs level in drinking water was 35.13 ng/L (the range of the concentrations of 4.49 to 174.93 ng/L), and the most commonly detected compounds were PFBA, PFOA, PFNA and PFOS [77]. Those authors also noted that the concentrations of analyzed compounds depended on the size of a city as it was higher in intermediate than in a large municipal agglomerations.

PFASs may also enter organisms through the skin and with inhaled polluted air, in which PFASs are present as a components of dust [78]. Short-chain PFASs may also come from degradation of branched fluorinated polymers [79]. Winkens et al. [80] measured PFASs in children’s bedroom dust and showed that the contents of PFASs was relatively high, while contents of PFASs precursors was insignificant. These results may suggest that the exposure via indirect swallowing of the dust (hand to mouth behavior) could play a significant role in total daily exposure to PFASs. This observation is supported by the studies demonstrating a positive correlation between the content of PFASs in dust present in shops (China), and the content of those compounds in blood of shopping assistants [81]. In industrial workers who were mainly exposed to PFASs by inhalation, the ratio of selected perfluoroalkyl compounds (PFOA, PFOS and PFHxS) in serum and plasma was 1:1 [82]. Studies conducted on ethnically heterogeneous populations, such as Boston-area Project Viva pre-birth cohort, the 2007/8 and 2009/10 National Health and Nutrition Examination Survey (NHANES) and Child Health and Development Studies pointed out on consistent differences in the concentrations of selected PFASs in blood dependly on race [83,84]. Harris et al. showed that the concentrations of PFASs were the lowest in children of black and other race mothers than in white mothers. Similar observations were made by Boronow et al. [84], who showed significantly lower PFOA and PFHxS levels in African and American women compared to non-Hispanic white women. Similar, in NHANES project lower PFASs concentration in Mexican Americans compared to native Americans have been reported. None of the discussed publication (that based on the above cohort studies) has proposed the molecular mechanism involved in this observation. However, the authors indicated that mainly sociodemographic, behavioral and health factors as well as maternal PFASs level (measured during pregnancy) may influence the amount of PFASs in blood. Harris et al. [83] listed the main predictors for elevated PFASs concentrations in the blood of children, such as older child age, lower adiposity, carpeting or a rug in the child’s bedroom, higher maternal education and higher neighborhood income. Some authors have emphasized the relationship between the mother’s higher education with the possibility of obtaining a higher monthly income on this account, and an increased concentration of PFASs in the blood serum. It can therefore be suspected that the increased exposure to PFASs may be associated with an increased standard of living, which is followed by a higher level of consumption, including more frequent manifestations of risky behaviors, such as repeated consumption of fast-food foods per week [83,85,86]. Other behaviors that conducive to exposure to perfluorinated compounds, include eating food in cardboard packaging, furnishing the interior with modern carpets and stain-resistant furniture, or even the regular use of dental floss [84].

Siebenaler et al. [87] in their study on a group of 37 young volunteers (over 18 years of age) demonstrated a correlation between exposure-favoring behavior and increased serum PFHxS level (1.07–12.55 ng/mL) that was surprisingly higher compared to PFOA (0.3–4.07 ng/mL). Contrary, studies on isolated human epidermis demonstrated that approximately 24% of the PFOA was absorbed through all layers of skin within a day, and that over 40% of the absorbed dose was accumulated in the skin [88]. There is limited evidence that PFASs pass the blood–brain barrier. Harada et al. [89] demonstrated that concentrations of selected PFASs in the cerebrospinal fluid was much lower than their serum level. On the other hand, it was shown that PFASs levels in brains of rat and mouse fetuses were higher compared to their concentrations detected in maternals brains [90,91].

### 3.2. Biotransformation and Accumulation

Perfluoroalkyls are not biotransformed in living organisms, and therefore their distribution should not be significantly different depending on different exposure routes. It is believed that high PFASs bioretention is associated with four factors. Firstly, easy and effective resorption of these compounds in the alimentary tract and in the respiratory system results in their high bioaccumulation potential. During exposure to PFASs over 90% of their amount is absorbed in the organism via diet and respiratory routes within 25 to maximally 90 min [69]. Secondly, strong binding to plasma/serum proteins (mostly to albumin), which determines presence of a specific reservoir of PFASs in the organism. There are reports (in vitro study) showing that over 99% of the PFOA, PFBS, PFHxS, and PFOS fractions could be bound to bovine serum albumin [92]. Interestingly, it was found that PFSAs had a slightly higher protein binding affinity, compared to PFCAs with the same carbon chain lengths. Some studies have also indicated that most PFASs had a stronger affinity to albumins than for other plasma proteins [92,93]. Spectroscopy and molecular modeling research of Liu et al. [94] showed that strong binding potency of PFOS, PFHxS or PFBS to human serum albumin is the result of the characteristic structure of this protein, which easily allows to create bonds, mainly through electrostatic forces and hydrogen bonds.

PFASs do not undergo a classic biotransformation, and their elimination is inefficient [69]. Studies conducted by Maestri et al. [95] and Peng et al. [96] have demonstrated that PFASs are preferentially accumulated in blood, lungs and kidneys. Similar observations were made by Pérez et al. [97], who analyzed postmortem human organs for PFOA content. Interestingly, the study demonstrated an unusually high content of several PFASs in bones (from 0 to 234 µg/kg). In the study of Koskela et al. [98] (2017) a potential association between PFASs impact on bone microarchitecture of human femoral bone samples and differentiation of human osteoblasts and osteoclasts was observed. It was revealed that those chemicals caused an increased resorption in osteoclasts. Bone tissue is highly differentiated, consists of osteoblasts and osteoclasts and is continuously remodeled via resorption by hematopoietically derived osteoclasts and finally formed (rebuilt) by mesenchyme-derived osteoblasts. Disruption of any of the above processes can lead to pathological changes in the structure of bone tissue, which may finally lead to increased fracture risk and bone diseases, such as osteoporosis. It has been proven that there is a certain association between PFHxS concentration and bone mineral density in patients suffering from osteoporosis [75].

Perez et al. [97] also reported a higher accumulation of PFOA in the liver, whereas PFHxS and PFBS were preferentially accumulated in kidneys. Interestingly, a correlation between sex and the level of bioretention of selected PFASs was observed, which indicated that accumulation of those compounds could be as much as three times higher in females, compared to males [99].

### 3.3. Excretion

PFASs are eliminated from the human organism mostly with bile, and also with urine. Trace amounts are also eliminated with feces and breast milk [23,89]. It was demonstrated that the elimination ratio of individual compounds belonging to the group of PFASs depended on the type of absorbed compound, species of the exposed individual as well as its age and sex. A general tendency has indicated a more rapid elimination of PFASs by females [23,100,101], which may be explained by differences in renal activity, including differences in activity of proteins transporting organic anions [102]. Furthermore, rapid elimination of PFASs by women may be partially explained by PFASs offloading into menstrual blood, as well as in childbirth [103]. According to Wong et al. [103] research models, menstruation reduced the time of PFOS elimination by over eight months. In the studies on the pharmacokinetics of the removal of PFASs from the human body, there is no evidence of a relationship between a relatively higher concentration/accumulation of PFASs in women and the rate of removal of these compounds from their body.

In a study conducted on a group of 86 people, it was demonstrated that urinary levels of selected PFASs (PFOS and PFOA) were positively correlated with their blood levels [23]. A correlation was not demonstrated only for perfluoroundecanoate (PFUnA). Moreover, efficacy of elimination was reduced with elongation of the carbon chain and its linearity (branched isomers were more easily eliminated). PFHxS (C6) was an exception from this rule, because its elimination was slower than the elimination of PFOS. The authors also demonstrated that PFCAs were more efficiently eliminated from the organism, compared to PFSAs with the same number of carbon atoms in the chain.

It was found that breastfeeding time was positively correlated with the serum PFASs level in children [102]. At the same time, breastfeeding was found to be one of the methods of elimination of PFASs from females, as serum PFOS and PFOA levels in breastfeeding women were lower than in non-breastfeeding women [104]. No similar correlation was demonstrated for PFHxS, in case of PFBS the assessment has not been done.

The PFASs half-life in human organism is variable (Table 4) and ranges from several hours (i.e., for PFBA) to as much as 15.5 years (for PFHxS) [105,106]. Interestingly, other studies showed that PFHxS elimination time was 5.3–7.3 years [8,107]. Nevertheless, the time is long, which may be explained by permanent exposure to relatively low levels of the compound and slower elimination processes [101].

In 2018, the European Food Safety Association (EFSA) evaluated the tolerable daily intake (TDI) valid at that time and determined the tolerated weekly intake (TWI) at the level of 13 and 6 ng/kg/week for PFOS and PFOA, respectively [109].

## 4. PFASs in Environment

### 4.1. Abiotic

PFOA and PFOS are two PFASs most commonly used in the industry and the most commonly determined PFASs in living organisms and the environment. Their prevalence and long-distance migration are evidenced by data regarding their presence in soil [110,111]; air [112], including Arctic air [113]; groundwater [114]; deposits [115]; snow in regions, including the Antarctic [116]; and rain water and surface water [117]. Due to their amphophilic properties, PFASs relatively easily migrate to the aquatic environment [65]. Significant concentrations of PFOA and PFOS in high-mountain glacier in Tibet [118] and of PFBA in snow in the Arctic region (Devon Ice Cup) [119] may be explained by easy geographic shift of those compounds from highly polluted areas. Moreover, significantly higher concentrations of short-chain PFASs in samples of water collected from streams of selected rivers was observed [120,121], which demonstrated the increased role of those substances in the chemical industry concentrated near water courses. The mean PFBA, PFHxA, PFBS and PFHxS concentrations determined in streams around the Bohai Sea in China was 11.3, 72.5, 52.3 and 333 ng/L of water, respectively [121].

The content of 17 perfluoroalkyl substances was also determined in the form absorbed on the surface of less than 10 µm air bubbles (PM10). A study performed in coastal cities of the Bohai and the Yellow Seas [122] demonstrated that detectability of short-chain compounds, such as PFSAs (C5) was higher compared to other long-chain PFASs. The atmospheric concentrations of perfluorinated compounds are highly variable. For example, the total concentration of 13 perfluorinated substances (including PFOS, PFOA and PFHxS and other PFASs) in air samples collected in the Greater Bay Area (China) was 122 ± 41.5 pg/m^3^, while PFOS was the dominant contaminant in the atmosphere [123]. Importantly, PFHxS was detected with frequencies of 83%. Similarly, Seo et al. [124] demonstrated significant contribution of short-chain PFASs, PFBS and PFHxA, in air pollution in Korea.

A high level of pollution with perfluorinated compounds is also determined in the areas of fire-fighting tests, and this is associated with migration processes of components of fire-extinguishing foam into soil through infiltration and desorption, further intensified by rain. Detectability of PFOS at 100% in water or soil samples, at the highest levels among all analyzed PFASs, was demonstrated in numerous studies of areas where fire-fighting actions have been or are carried out (airfields and training grounds) [125,126,127].

It was found that the exposure to PFASs is intensified also by presence of dust (on which compounds settle) in homes, offices and industrial rooms. Dust collected from 65 children’s bedrooms in Finland (in 2014/2015) demonstrated accumulation of nearly 53 compounds belonging to the group of PFASs [76]. It was estimated that PFASs concentrations in the dust of houses and public facilities (like a library and a shopping mall) in Korea ranging between 29.9 and 97.6 ng/g [128] while PFOS content in house dust in Germany (Bavaria) and Spain (Catalonia) was 97.1 ng/g and 3.51 ng/g, respectively [129,130].

### 4.2. Biotic (Human)

Despite successive withdrawal and limitation of industrial use of long-chain PFASs, their levels determined in various components of the environment and blood of the general population, remain significantly high. It could be related with high environmental stability of PFASs but also some exclusions to the B list of the Stockholm Convention, allowing further use of PFOS and PFOA (in certain concentrations) in selected products Maintenance of limited concentrations of PFASs in the production of everyday-use products, such as packages for fast-food, kitchenware and air fresheners, leads to common and inevitable exposure to these compounds.

The level of PFOA and PFOS in blood of the general population was measured in many countries: USA, China, European countries (Poland, Germany, Czech Republic, Belgium, Italy), Brazil and Korea [22,59,131]. Blood levels of PFOS and PFOA were different, reaching as much as 33.1 and 4.5 ng/mL, respectively [22], while in the serum of occupationally exposed workers, they reached values of 490 and 5100 ng/mL, respectively. The studies of Benford et al. [59] demonstrated the presence of these compounds in high concentrations in workers exposed to PFASs. For PFOA the serum level reached several hundred mg/L. The highest concentration of PFOA in blood of the general population was found in South Korean women. The mean measured level was 88.1 ng/mL of serum (range: 15–256 ng/mL) [131]. The study conducted on 37 young American volunteers (26 women and 11 men) aged between 22 and 34 years showed the presence of the mean PFOA, PFOS and PFHxS in their blood serum, in the concentrations of 1.57, 4.96, and 3.17 ng/mL, respectively [87]. Table 5 and Table 6 present mean PFASs levels determined in the general population (including serum of pregnant women and umbilical blood) and occupationally exposed workers. Analyses of PFASs content in blood serum of mothers and children (three and eight years old) demonstrated that those compounds were present in the following decreasing order of the concentrations: PFOS > PFOA > PFHxS > PFNA [132]. The presence of PFOS and PFOA at the top of this hierarchy is probably related to the long-term occurrence of these compounds in the environment, because they have been producing for over 70 years [6] and additionally they are characterized by incomparable longer half-life in human blood (up to 27 years) [108]. The concentration of PFHxS and PFNA determined in the serum of mothers and children was lower, because these compounds are eliminated from the body relatively faster [108]. Moreover, their use in the industry is much shorter, and thus general population is exposed to these compounds for a shorter time. Furthermore, the higher concentration of PFHxS than PFNA is probably the result of wider use of this compound in the industry as well as more frequent use of this substance as an alternative to PFOS. This was also reflected in an increase of PFHxS levels in blood of general population during several years after reduction of the production and use of PFOS [26]. It is believed that the concentrations of short-chain PFASs are greatly lower compared to long-chain ones. For example, the levels of PFHxA, PFBA and PFBS determined in the general population of Germany were less than 1 nM [133,134].

## 5. Endocrine Disruption Caused by PFASs

According to World Health Organization definition, EDC is a compound that alters function of the endocrine system and causes adverse health effects in an intact organism [1]. Under the term “adverse effects” are disturbances in metabolism, reproduction, hormone-dependent cancers or deregulation of the immune system. For a better understanding of the mechanisms involved in deregulation of endocrine system, these adverse effects are also considered in this chapter.

There are two major potential molecular mechanisms of endocrine harmful effects caused by PFASs: impact on steroidogenesis and interaction with nuclear hormone receptors.

This section describes the potential mechanisms of action along with the endocrine effects caused by PFASs.

### 5.1. Influence on Steroidogenesis

**Figure 2:**
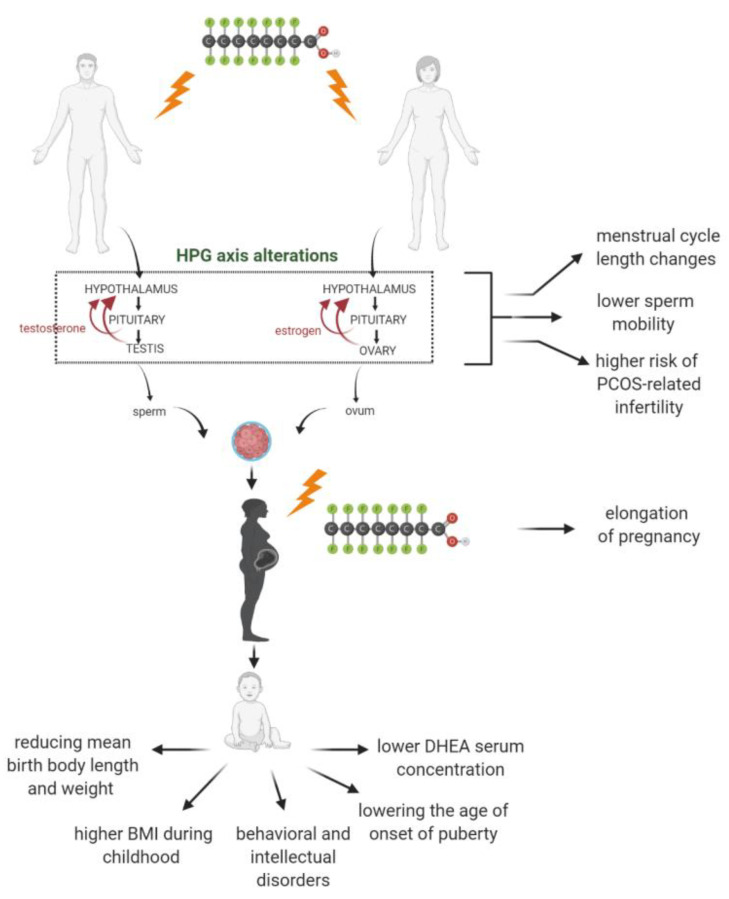
Alterations in hypothalamic–pituitary–gonadal axis functions with potential health effects caused by per- and polyfluoroalkyl substances (PFASs) exposure [32,44,46,151,152,153,154].

Considering numerous reports confirming the effect of PFASs on activation of PPARα, indirectly associated with changes in lipid metabolism, including bio-synthesis of cholesterol, the effect of PFASs on the metabolism of steroid hormones cannot be excluded [143]. Analysis of steroidogenic effects of PFOS in the human adrenocortical carcinoma cell line (H295R) have shown alteration in the expression of several genes involved in the biosynthesis of steroids (increasing expression of *3βHSD2*, *CYP11B2* and *CYP19*; decreasing expression of *CYP17*, *17βHSD4*) [144]. Moreover, the same research team confirmed alteration in synthesis of estradiol and progesterone in this cell line after PFOA exposure. In a similar study published in 2016 [31], conducted on the same cell line (H295R), those authors also indicated that two of the most widely used PFASs, i.e., PFOA and PFOS, induced transcription of two genes (*CYP19* and *3βHSD2*) involved in sex hormones synthesis. Furthermore, the qPCR analysis revealed that PFOS led to the transcriptional induction of *CYP112B*. The results suggest that PFOS and PFOA alter the pathway of aldosterone synthesis in adrenal glands, and this may affect the water and mineral balance in living organisms. In contrast, Behr et al. [145], in the H295R steroidogenesis assay, found that PFOA only slightly increased progesterone secretion and, similar to PFOS, slightly decreased estrone secretion. Moreover, observed effects occurred only at the concentration of above 10 µM, which may suggest that tested PFASs of long- (PFOS and PFOA) and short-chain (PFHxS, PFHxA and PFBA) might not cause human hormonal system disorder during everyday human PFASs exposure. The aim of cohort study (Odense Child Cohort) performed on 373 maternal–child pairs was done to determine the endocrine disruption potential of both, long- (PFOS and PFOA) and short-chain PFASs (PFHxS, PFNA and PFDA) [146]. Obtained data showed that prenatal exposure to PFDA during two-fold increase concentration of PFDA in maternal serum predisposed infants to significantly lower serum level of important for development steroid hormone precursor, dehydroepiandrosterone (DHEA). DHEA is one of the most abundant circulating steroid hormones in humans, a large reservoir of precursors for intracellular production of androgens and estrogens in non-reproductive organs/tissues [147]. DHEA modulates main cardiovascular signaling pathways, anti-inflammatory response and play an important role in brain development and physical maturation [148]. Moreover, it was found that fetal exposure to PFDA probably predispose infants at mini puberty (at four months of age) to a lower level of other steroid precursors—androstenedione and dehydroepiandrosterone sulfate (DHEAS). Observed effects were characteristic only for female infants, while, in the case of males, such correlations have not been demonstrated, and they could be associated with that females depend more significantly on adrenal androgen production than males. The study based on Odense Child Cohort [146] did not show association between PFASs concentration in maternal blood and the level of follicle stimulating hormone (FSH) and luteinizing hormone (LH). Interestingly, these data are in line with the findings of another (Danish) prospective study that have also reported no association between prenatal PFOS and PFOA exposure and incidents of the higher FSH and LH levels at later age [149] or alterations in testosterone serum level in young men [150]. Interestingly, another Danish prospective study [151] showed that prenatal exposure to PFOS, PFHxS, PFNA or PFDA is associated with lowering the age of onset of puberty. The effect observed for PFDA and PFNA was weaker and the mean age of onset puberty was higher than for other analyzed PFASs. Alterations of pubertal development induced by PFASs may be related with disturbance of hypothalamic–pituitary–gonadal axis functions. Similar effects were reported in the study performed on laboratory animals [32]. Alterations in hypothalamic–pituitary–gonadal axis functions, with potential health effects, are shown on Figure 2.

#### Cholesterol Homeostasis Alterations

Cholesterol is a 27-carbon polycyclic lipid, which is the precursor of all steroid hormones. De novo synthesis of cholesterol (approximately 1 g per day) takes place mainly in the liver [155]; therefore, some disturbances in hepatocytes function may potentially contribute to cholesterol synthesis disorders. It is noteworthy that alterations of lipid metabolism during peroxisome, proliferator-activated receptor gamma or/and alfa (PPARγ, PPARα), retinoid x receptor (RXR) and the mRNA modulations can also be crucial for cholesterol homeostasis in the case of deficit of specific substrates for cholesterol synthesis or regulation of plasma cholesterol levels [156,157]. Another important element of cholesterol distribution plays transcriptional regulation modulated by nuclear receptors, such as liver X receptor (LXR) and farnesoid X receptor (FXR) [158].

Several epidemiological studies [159,160,161] have reported positive associations between PFASs (PFOS and PFOA) serum concentrations and serum levels of cholesterol. Interestingly, findings obtained in human study were opposite to studies performed on animals. In the experiments conducted on monkey and rats PFASs exposure caused an increase in serum cholesterol and triglyceride levels, while in epidemiological studies, a decrease of these parameters has been observed [109,162]. Interestingly, a study on mice (APOE*3-Leiden CETP) with similar to human lipoprotein metabolism model showed that PFOA induced a decrease of total cholesterol and plasma triglycerides levels inhibited [163]. It should be underlined that the concentrations of PFOA analyzed in this study were much higher than those determined in general population exposure. Data published in 2020 [164] revealed that PFOA, PFOS and PFNA increased triglyceride levels and inhibited cholesterogenic gene expression in HepaRG cells.

**Figure 3:**
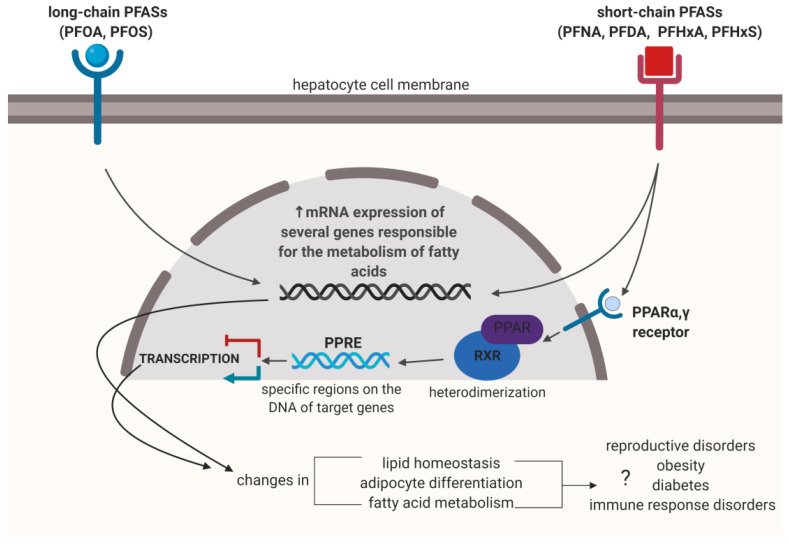
Potential molecular mechanism of PFASs hepatotoxicity. In liver cells, PFASs activate peroxisome-proliferator-activated receptor (PPAR), which induces heterodimerization with retinoid x receptor (RXR). Complex binds to specific sequence of DNA–PPREs (peroxisome-proliferator hormone-response elements), which occurs in the promoter region of a gene and modulate transcription. Based on papers of Reference [170].

Studies published in 2011, by Florentin et al. [165], demonstrated that both PFOA and PFOS had a cytotoxic effect on human HepG2 cells but did not cause DNA damage nor micronuclei formation in tested cells. Papers published later demonstrated that PFOA, PFOS and short-chain PFASs, PFNA, PFDA and PFHxA increased the expression of mRNA of twelve genes associated with metabolism of fatty acids, and several of these genes were induced in the liver [152]. Similar observations were made in mice treated with PFASs (PFOA, PFNA and PFHxS), where the expression of genes responsible for both decomposition and synthesis of lipids was demonstrated [166]. Authors of the study suggested that the disturbance of the expression of genes responsible for regulation of lipid homeostasis is the basic mechanism of hepatotoxicity. That hypothesis may be supported by the results obtained by Rosen et al. [167]. The study demonstrated that short-chain PFASs, such as PFNA and PFHxS, activated the PPARα transcription factor receptor, which led to increased metabolism of fatty acids, increased apolipoprotein I (apo A1) level. Moreover, an increase of the high-density lipoprotein (HDL) fraction and reduction of lipoprotein lipase (LPL), leading to a reduced level of serum triglycerides and cholesterol transfer disturbance was noted [168]. Importantly, it was also demonstrated that the short-chain PFHxS caused an almost 10-fold stronger expression of the oxidoreductase, one of regulators of lipid metabolism, and stearoyl coenzyme A desaturase (Scd), compared to PFOA, PFOS and PFNA [169]. Moreover, it was proved that PFBS stimulated development of adipocytes (adipogenesis) in 3T3-L1 cells [170] by activation of the mechanism intermediated by extracellular signal-regulated kinases MAPK/ERK (mitogen-activated protein kinases). It seems that induction of adipokinesis starts in receptors on the cellular surface, from where the signal is transmitted to the nucleus (Figure 3). 

### 5.2. Hormones Disturbance

Hormone-like properties of substances classified as EDCs are often based on the ability of binding to specific receptors and the nuclear receptor superfamily, which are the most abundant classes of transcriptional regulators [171]. Molecules of EDCs connect mainly with specific receptors, such as estrogen receptors (ER), androgen receptor (AR) or thyroid hormone receptor (TR), which regulate development, reproduction and metabolism. One of the most popular EDCs interacting with all three receptors is BPA [172,173,174].

**Figure 4:**
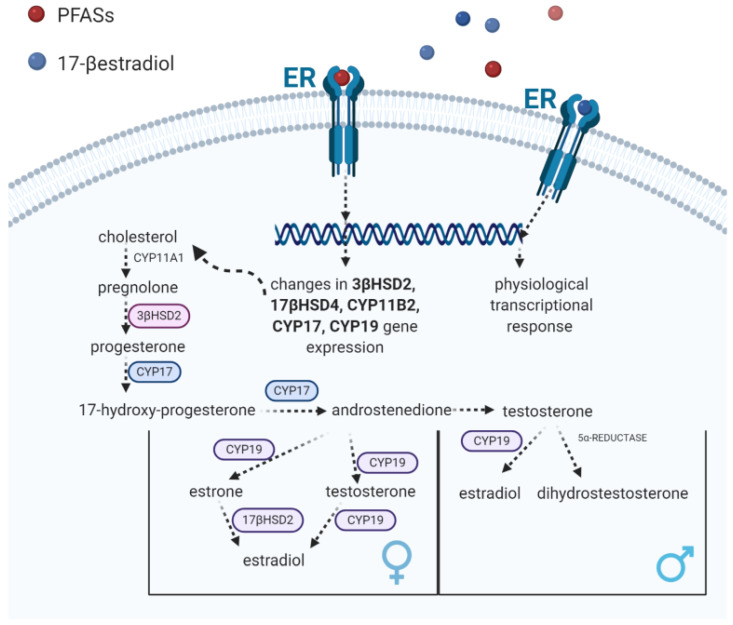
Transcriptional regulators of the cholesterol transformations via ER in male and female–implications caused by PFASs. ER, endocrine (nuclear) receptor.

Androgens are a type of steroids produced by the ovaries, testis, placenta, brain, glands and skin [175,176]. Androgen production is dependent on producing organs, and in the ovaries, this process is overridingly regulated by hypothalamus. Hypothalamus, which releases gonadotropin, stimulates of the pituitary gland towards LH and ADH secretion. In the next step, LH stimulates ovarian theca cells to transform cholesterol to two key hormones, testosterone (T) and androstenedione (A4), which mostly are converted to estrogens (17β-oestradiol, E2) in female. Testosterone in male is synthetized mainly in testis in Leydig cells, and is converted to 5α-dihydrotestosterone (DHT). The stimulation of T, DHT or E2 is regulated via the nuclear receptor superfamily of ligand-activated transcription factors, mainly AR and ER. There are two predominant isoforms of ER: estrogen receptor α (ERα) and estrogen receptor β (ERβ). ERs respond to 17β-estradiol during physiological signaling pathways [177] (Figure 4).

The study on CV-1 cells and the MDA-kb2 cell line indicated that PFOS, in concentration-dependent manner, interact with ERα and TR disrupting estrogen and thyroid hormone functions [178]. Similar results were obtained in mechanistic assays for human ER, where PFOA, PFOS, PFNA and PFDA exposure significantly enhanced human ERα-dependent transcriptional activation [179]. An in vitro study of PFOS and PFOA effects in stimulated porcine ovarian cell (theca and granulosa cells) also confirmed inhibition of estradiol and progesterone secretion [180]. Interestingly, in vitro study on human cell lines (MCF-7, LNCaP, MDA-kb2 and H295R) demonstrated weak or no estrogenic and androgenic activity of PFASs in tested cells and their slight impact on steroid hormone secretion [178]. Opposite, numerous studies confirmed influence of PFASs in binding inhibition T to AR. It was demonstrated that PFOA and PFOS act as antagonist to AR in HeLa cells [181] what is in line with findings confirming affinity of PFOA, PFOS, PFHxS, PFNA and PFDA to ER and AR [182]. These significant differences may arise from application of different xenobiotics, exposure time, concentrations, different cell lines or reporter plasmids used in the experiments. It is worth noting that simultaneous incubation of PFOA at concentration determined as a result of occupational exposure (10 µM) with T (in physiological concentration of 10 nM) contributed to reduction of AR nuclear signal by nearly 20% [181,183]. Moreover, it was reported that the binding affinity of PFASs with the same number of carbons in chain is dependent on terminal groups and decreases as follows: sulfo group > carboxylic acid group > alcohol group [184].

Thyroid hormones (THs) play critical roles in almost all physiological functions of nucleated cells, including a crucial role in mammalian brain development. Low THs levels at critical steps of neurodevelopment can lead to intellectual and behavioral disorders.

Neurological deficiencies and disorders in behavioral and/or intellectual development as a result of exposure to PFASs are discussed in the subsection “Neuroendocrine toxicity”.

Available studies have indicated the effect of PFASs on the human hormonal system through various pathways and mechanisms, i.e., enhancement of the thyroid feedback loop [185]. Changes in those pathways and mechanisms may potentially result in observed alterations in levels of selected hormones, including thyroid stimulating hormone (TSH), total thyroxine (T4) and free thyroxine (FT4) [186,187,188]. The level of short-chain PFNA is positively associated with the free T4 level in pediatric population (Korea) [189]. In the cohort study carried out in the USA (as a part of the Viva project) the level of maternal thyroid hormones (TSH and FT4) and blood levels of six PFASs, including PFOS, PFOA and PFHxS were measured in 732 women and 480 neonates [190]. The results confirmed the effect of prenatal exposure to PFASs on the thyroid function of mothers and infants.

What is important, the effects of PFAS exposure on thyroid hormone homeostasis differed between sexes and observed associations were stronger for women. It was demonstrated that there are associations between serum PFOS level and increased thyroid stimulating hormone and positive association between repeated measures of serum PFNA and total T4 level in women [41]. It was also found that PFASs caused stronger disturbance in women thyroid hormone homeostasis—PFOA and PFNA levels were positively associated with TSH concentrations and significantly associated with free triiodothyronine (T3) concentrations in serum [42].

It was revealed that PFASs (including PFOS, PFOA, PFBS) competed for binding sites in transthyretin (TTR, thyroid hormone transport protein) with T4. Importantly, the strongest affinity to TTR demonstrated short-chain PFASs, PFHxS, even stronger than PFOS and PFOA. However, it should be added that binding potency of PFBS to TTR was approximately 12-times lower than binding potency of T4. Meta-analyses of 20 articles investigating association between PFASs exposure and thyroid function in humans found that blood PFOS level positively correlated with free T4 level, while PFHxS showed a negative correlation with total T4 level [191].

A greater attention is drawn recently to the structural specificity of PFASs. It turns out that higher levels of PFHxS and of branched forms of PFOS determined in blood serum of pregnant women were associated with higher levels of TSH and/or lower levels of FT4, compared to changes observed in case of the exposure to linear forms of those compounds [192].

### 5.3. Fetus and Newborn

Considering the fact that vast a majority of available studies have focused on the effect of PFOA and PFOS on living organisms, the conclusions on toxicity of PFASs are drawn mostly on the basis of their results. As for the effect of PFASs present in blood serum or plasma of pregnant women on changes of weight or length of neonates, data are scarce and inconsistent.

**Figure 5:**
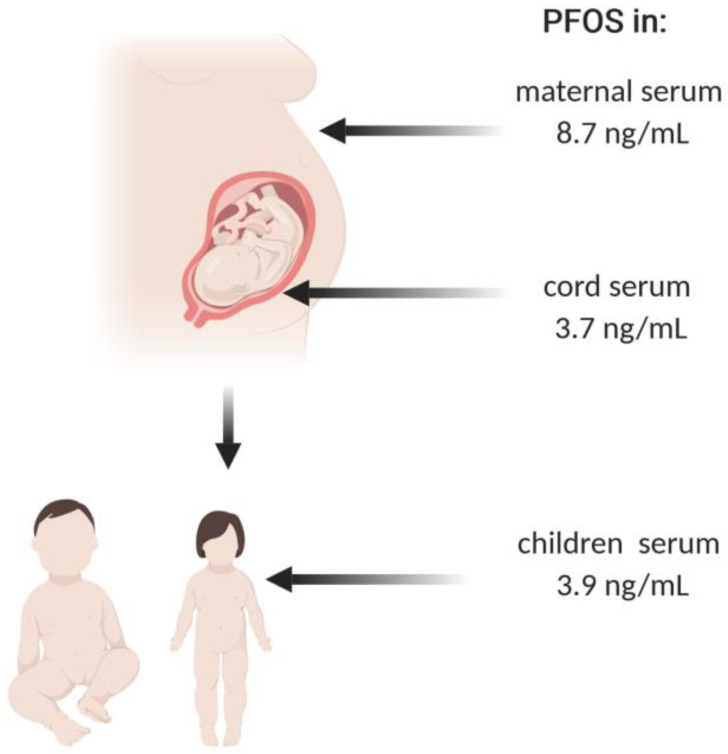
Concentrations of PFOS (mean) in maternal, cord and children serum [136,193]. The blood–placenta barrier limits the penetration of PFASs to the fetus. In the first years of life, the concentration of PFASs in the serum starts to increase.

Based on measurements of concentrations of selected PFASs in maternal blood, umbilical blood serum and neonatal blood, it has shown evident that PFASs are able to pass the placenta barrier [193]. Table 6 and Figure 5 shows different levels of PFASs determined in human serum from the blood of mothers and umbilical cord blood. Transplacental transfer efficiency (TTE) is in that case calculated as the quotient of the concentration of the compound in the blood or serum by determined maternal PFASs level. The comparison of TTE values for individual PFASs suggests that PFSAs penetrate the blood–placenta barrier less efficiently than PFCAs, which is associated with a greater affinity of PFSAs to serum albumins [194,195]. It is believed that the affinity of PFASs to plasma proteins determines TTE by limited availability of free PFASs molecules in blood [195,196]. Protein binding, lipid solubility and pKa values determine uptake of xenobiotics by fetus, and in case of PFASs, pKa values predispose them to easier blood–placenta transfer. Interestingly, fetal pH value may play a significant role in transfer of a compound to fetus and achievement of a xenobiotic/drug equilibrium. In physiological conditions fetal pH values range between 7.32 and 7.38, whereas maternal pH value is 7.38–7.42, and transport of all substances follows the difference of their concentrations. In pathological conditions, when fetal pH value is shifted towards acidic, blood–placenta transfer of xenobiotic becomes intensified by deprotonation of the free form of a xenobiotic into its ionized form (so-called: ionic entrapment) [197,198].

An interesting study was carried out by Spratlen et al. [203]. They analyzed the content of perfluorinated compounds in umbilical blood of children whose mothers were affected by the World Trade Center (WTC) catastrophe (umbilical blood and maternal plasma were collected between December 2001 and June 2002). Women living in the area of the radius of 2 miles around WTC buildings were included in the study. The research noted higher levels of PFASs, and particularly of PFOA (up to 13%), compared to the reference group, indicating necessity of further studies on the population of children exposed during their fetal life on higher concentrations of PFASs in that area.

Interestingly, the literature data indicate that a higher level of PFNA in umbilical blood is found in mothers with higher education [138]. This is the most probably associated with their lifestyle (living in bigger cities and using processed food). In their meta-analysis of bio-monitoring studies (internal exposure to PFASs associated with descriptors of social and economic status). Buekers et al. [204] concluded that education level could not be analyzed because of excessive range of descriptors themselves. However, they demonstrated that increased income was associated with internal exposure to PFOA, PFOS and short-chain PFASs such as PFNA and PFHxS.

Studies have demonstrated that PFOS, PFOA and PFHxS (both linear and branched) are able to penetrate from maternal serums, via umbilical serum, to the placenta [193]. A correlation was also demonstrated between the exposure of pregnant women to PFOA and reduced birth weight (cohort study on 1400 Danish women in 1996–2002) [151]. An increase of exposure to PFOA by each 1 µg/L resulted in reduced body length by 0.069 cm and reduced abdominal circumference by 0.059 cm. Cohort study on Danish mothers and their children was repeated in 2008–2013 [153]. The study demonstrated a significant reduction of the mean birth weight in the group of children exposed during the pregnancy to PFHxS at the concentration over the lowest quartile of exposure. Similar observations were made by Chen et al. [205]. Those authors demonstrated a correlation between umbilical blood PFOS level and probability of preterm birth and low birth weight of neonates (interestingly, the correlation was not noted for PFOA). Furthermore, cohort study carried out in Spain in 2003–2008 [202] also demonstrated that neonates born by mothers with plasma PFOA, PFNA and PFHxS levels twice as high as in the control group had their birth weight statistically reduced by 8.6–10.3 g. Similar observations have been made by another researches [206,207]. Interestingly, Manzano-Salgado [202] also demonstrated that the association between the PFOS level in maternal blood and reduced birth weight (although not statistically significant) was sex-specific (more pronounced changes in the case of the female sex). The effect of PFASs on duration of pregnancy have also been reported [153]. On the other hand, Gylennhammar et al. [201] indicated no correlation between birth length or weight (standard deviation score, SDS) and presence of PFASs in maternal serum. Interestingly, this correlation was demonstrated in another study, for older children, over the age of three years, stressing that high BMI during childhood favored obesity during adulthood [154].

Based on the analyses of the available scientific literature, there are no studies that have described a possible relationship between PFASs exposure and fetal aneuploidy.

### 5.4. Reproductive Toxicity 

The study on human spermatozoids demonstrated no genotoxic effect of PFOS, PFOA, PFNA and PFHxA [208]. Molecular mechanisms of PFASs reproductivity are poorly known, and the number of publications reporting their effect on the human reproductive system is limited. Analyses with the use of the innovative in vitro model of spermatogenesis carried out on stem cells indicated that PFASs (PFOS, PFOA and PFNA), independently and as a mixture, caused no changes of transmembrane mitochondrial potential, did not intensify reactive oxygen species (ROS) generation and did not decrease viability of spermatogonia, spermatocytes (primary and secondary) or spermatidia [209]. However, this study demonstrated that selected PFASs could have a long-term effect on fertility by exhaustion of spermatogonia pool and by disturbance of function of primary spermatocytes caused by reduction of expression of markers specific for these cells. The study conducted in China and Taiwan, which assessed potential link between selected PFASs exposure and sperm mobility revealed negative correlations between PFBS and other PFASs blood levels and mobility of sperm [210]. Interestingly, absence of reproductive toxicity in laboratory animals (Sprague Dawley and Wistar rats) after PFHxS exposure was confirmed [34,211].

### 5.5. Obesity

Three independent studies have demonstrated a correlation between prenatal exposure to PFASs and the risk of obesity in children and adolescents aged from 3 to 20 years [212,213,214].

Study published in 2020 found no consistent link between maternal serum levels of long-chain PFASs (PFOS and PFOA) and short-chain PFASs (PFHxS and PFNA) in early pregnancy and overweight in the children at four years [215]. On the contrary, the research conducted on overweight and obese Hispanic children found correlation between PFOA and PFHxS blood levels and alterations in glucose homeostasis, which was in line with children metabolomics profile [216]. Furthermore, Ram and Ducatman [217] found that PFNA level was positively associated with cholesterol and LDL levels in obese males and females.

### 5.6. Neuroendocrine Toxicity

It has been shown that prenatal exposure to certain EDCs can affect the neurodevelopment disorders: disturbance in synaptic connection and neuronal differentiation, alteration in neurotransmitter function or expression of genes involved in neuropeptide synthesis [218]. Interestingly, estrogen receptors are found to mediate many processes connected with these neuronal system disorders [219]. Such an effect was demonstrated for BPA, organophosphorus peptides pesticides and phthalates, which caused brain development as well as behavioral and cognitive disorders [220,221,222]. Similar reports might be referenced for PFASs. Epidemiological data describing the effect of PFASs on the nervous system, similar to others toxic effects caused by these compounds, are inconsistent and, in some cases, even surprising. Studies analyzing association between prenatal exposure and impulsive behavior of three-, seven- and eight-year-old children (based on the serum PFASs levels in mothers and children) mostly have demonstrated no significant correlation between these factors [223,224,225]. However, it was demonstrated that high (prenatal or/and at later age) exposure to PFNA which was associated with the higher serum concentration of these compound in five- and seven-year-old children can be associated with certain neurobehavioral disorders [225,226]. Moreover, it was found that higher serum levels of PFNA and PFOA in eight-year-old children may be associated with impairment of their executive reactions [226]. On the other hand, the study published in 2018 demonstrated a potential (interestingly-a positive) effect of perfluorinated compounds on the reading ability by three-, five- and eight-year-old children who had also been exposed to PFASs (PFOA, PFOS and PFNA) during the prenatal period and in early childhood [132]. The study of cognitive function of children who had been previously exposed to discussed compounds demonstrated a possible effect of PFASs on reduced motor skills, but also suggested a possible association between high exposure to PFASs with higher cognitive skills [227]. No correlation was found between prenatal exposure and IQ of five-year-old children [228].

Studies on rats constitute a basis for the discussion on a potential mechanism of neurotoxicity involving astrocytes. The basic function of these cells is transmission of neuro-transmitters between them and neurons. For that reason, any disorders of astrocytes function may lead to impaired function of neurons. The in vitro experiment on a single culture of cerebral cortex astrocytes and combined culture of astrocytes with neurons of the primary hippocampus of rat demonstrated that exposure to PFOS caused reduction of the glutamate synthase enzyme activity, reduced expression of genes of that enzyme, changed extracellular levels of glutamate and glutamine in astrocytes, induced apoptosis and autophagia and altered redox status in neurons [229]. The experiment performed by Wang et al. [230] demonstrated that d-serine from astrocytes could mediate apoptosis of those cells caused by PFOS. An increased expression of the gene and protein of the N-methyl-d-aspartate receptor (NMDAR) subunit in neurons associated with these astrocytes was also demonstrated. Therefore, this study contradicted with conclusions drawn from the experiment of Li et al. [229] postulating a soothing effect of astrocytes on PFOS-associated neuron function disorders. An attempt was made to explain the mechanism of PFOS toxicity on neurons during the fetal development. For this purpose, changes and role of expression of glutamate receptor 2 (GluR2) in primary cortical neurons and brains of rat neonates exposed to PFOS were studied [90]. An interesting observation was made that despite the same level of PFOS in plasma and the liver of female rats and rat neonates, a 5-fold higher cerebral level of that compound was found in young individuals, combined with reduced expression of the GluR2 subunit and increased susceptibility to glutamate. An attempt was made to assess the effect of PFOS, PFBS and PFHxS on synaptic plasticity (also referred to as long-term potentiation-LTP, associated with, among others, learning processes) of hippocampus cells in rats, by acute injection of these compounds into cerebral cortex [231]. The study was designed to determine if exposure to PFOS and its replacements may affect the mechanism of opening the receptor channel of the NMDA-type glutamate receptor, and subsequent externalization of the AMPA-type glutamate receptors, accompanied by certain changes in cell morphology. It was demonstrated that PFOS, PFBS and PFHxS caused changes in LTP, but with no disorders in neurotransmission.

### 5.7. Genotoxicity, Cancerogenicity and Mutagenicity

At present, PFOS is classified in group three, according to the International Agency for Research on Cancer (IARC). Therefore, it is not considered carcinogenic for humans. In the scientific literature, there is only one report indicating existence of a correlation between urinary bladder carcinoma in plant workers and high level of exposure to PFOS [232]. No other publication that would support that observation appeared in the last years.

However, considering reports indicating a possible xenoestrogenic effect of PFASs, those compounds are suspected to favor development of breast cancer. The study published in 2020 [51] indicated that these discussed compounds could be a significant factor promoting this type of malignancy. The abovementioned cohort study was carried out in France, with 388 women, and demonstrated a linear relationship between the serum PFOS level and development of receptor-positive tumors. Interestingly, the study of another team of researchers, published one year earlier, contradicted that report and indicated only a marginal relationship between PFHxS and PFUnDA levels and breast cancer, and only in case of receptor-negative tumors [233]. On the other hand, in the study of the human hepatoma cell line (HepG2) a concentration-dependent increase of DNA injuries was demonstrated for cells incubated with PFOA, PFOS, PFHxS and PFNA [234]. Generation of ROS and DNA bases lesions, including 8-hydroxy-2’-deoxyguanozine (8-OHdG), is believed to be one of the basic mechanisms leading to PFASs-associated DNA damage. The study on the human lymphoblast cell line (TK6) confirmed a high genotoxic and oxidative potential of PFOA and PFNA. Interestingly, PFNA caused generation of 8-OHdG at lower concentrations than PFOA [235].

## 6. Conclusions

Findings for PFASs endocrine toxicity have been shown to be inconsistent, and that fact is related to some major limitations. Firstly, the limitations are obvious and follow from using different research models. Using different cell lines for research results in different sensitivities of these cells to the EDCs discussed. Moreover, it is impossible to compare disorders of the hormonal system in the fetus with the effects observed in sexually mature individuals. Secondly, most of the studies have been conducted by using only one selected compound from PFASs group, while the general population is simultaneously exposed to many xenobiotics from this group with endocrine potential. The interaction of that co-exposure may cause different molecular effects than those observed in the experiments with a single chemical. Thirdly, the available literature has suggested two commonly used methods for the explanation of obtained results—via an explanatory and via predictive-focused analyses. Each of these methods can lead to different conclusions for the same experiment and potentially generate inconsistent results.

Endocrine-adverse effects observed as an action of long- and short-chain PFASs not only depend on the concentration, chain length and type of a functional group of the compound, but also on age, sex and species of the exposed individuals. Short-chain PFASs (C4–C6) currently replacing long-chain compounds (particularly C8, including PFOA and PFOS) demonstrate a similar endocrine disrupting potential to their long-chain equivalent. Nevertheless, further studies are required to clarify the mechanism of PFASs endocrine toxicity.

## Figures and Tables

**Table 1:** The chemical structure of per- and polyfluoroalkyl substances (PFASs) most often used in the industry.

	Abbreviation	Number of Carbons in Fluorinated Chain	Chemical Names	Chemical Formula
PFASs	**PFCAs**			
	PFOA	7	perfluorooctanoic acid	C_8_HF_15_O_2_
	PFHxA	5	perfluorohexanoic acid	C_6_HF_11_O_2_
	PFNA	8	perfluorononanoic acid	C_9_HF_17_O_2_
	PFDA	9	perfluorodecanoic acid	C_10_H_19_O_2_
	**PFSAs**			
	PFOS	8	perfluorooctane sulfonic acid	C_8_F_17_SO_3_K
	PFHxS	6	perfluorohexane sulfonic acid	C_6_F_13_SO_3_K
	PFBS	4	perfluorobutane sulfonic acid	C_4_F_9_SO_3_K

PFCAs, perfluoroalkyl carboxylic acids; PFSAs, perfluoroalkane sulfonic acids; PFOA, perfluorooctanoic acid; PFHxA, perfluorohexane acid; PFNA, perfluorononanoic acid; PFDA, perfluorodecanoic acid; PFOS, perfluorooctanesulfonic acid; PFHxS, perfluorohexane sulfonic acid; PFBS, perfluorobutane sulfonic acid.

**Table 2:** Biological effects provoked by long- and short-chain PFASs.

Category	Study Type	PFASs	Results	References
Endocrine disruption	Odense Child Cohort (adults, *n* = 210)	PFOS, PFOA, PFHxS, PFNA, PFDeA	association between serum PFOS level and increased thyroid stimulating hormone; positive association between repeated measures of serum PFNA and total T4 level in women	[41]
	Cross-sectional (children, *n* = 85)	PFOA, PFNA, PFUnA, PFDA	disturbance of thyroid hormone homeostasis (differs between sexes)	[42]
Obesity	Cross-sectional (adults, *n* = 1612)	PFOA, PFOS and other PFASs	positive association between PFASs exposure with overweight and increased waist circumference (with particular emphasis on the effect of PFOA on selected obesity parameters)	[43]
	Odense Child Cohort (mother–child, *n* = 412)	PFOS, PFOA	each ln-unit increase in maternal serum PFOS and PFOA levels during pregnancy increased odds for overweight or/and obesity in children	[44]
	Odense Child Cohort (mother–daughter, *n* = 359)	PFOA, PFOS, PFNA, PFHxS	prenatal exposure to PFOA and PFOS was associated with girls % body fatness (except PFHxS and PFNA)	[45]
Diabetes	Cross-sectional (adults, *n* = 7904)	PFOA	serum PFOA was positively associated with diabetes in men; PFOA disrupt cholesterol metabolism (at environmental relevant level)	[46]
	Odense Child Cohort (adults, *n* = 4129)	PFOA	no association between PFOA exposure and incidence of diabetes	[47]
Reproductive disorders	Case-control (adult women, *n* = 367)	PFOS, PFOA, PFBS, PFHxS, PFNA, PFDA and other	association between plasma PFDA level and significantly increased risk of PCOS-related infertility	[48]
	In vitro (primary human placental cytotrophoblasts)	PFOS	apoptosis of human placental syncytiotrophoblasts)	[49]
	Odense Child Cohort (couples, *n* = 501)	PFOA, PFOS, PFNA, PFOSA, PFDeA and other	associations between two perfluoroalkyl substances and menstrual cycle length changes (2–5% shorter menstrual cycles during PFOA exposure and 3% longer during PFDeA exposure); association between selected perfluoroalkyl substances and lower pregnancy probability	[50]
Breast cancer	Odense Child Cohort (adult women, *n* = 388)	PFOS, PFOA	positive association between high concentrations of PFOS and breast cancer risk (for analyses that were restricted to expression of estrogen receptors: ER+/PR+ tumors)	[51]
Hepatotoxicity	Odense Child Cohort (adults, *n* = 1605)	PFOA, PFOS, their isomers and other	clinically significant hepatic cell dysfunction (abnormal liver function biomarkers: prealbumin and ALT level)	[52]
	Cross-sectional (adults, *n* = 1016)	PFOA, PFOS, PFOSA, PFNA, PFDA, PFHxS and other	positive association between the changes in activity of ALT, ALP, and GGT after PFASs exposure and changes in circulating bilirubin level	[53]
	Cross-sectional (adults, *n* = 30,723)	PFOA	association between PFOA and ALT, a marker of hepatocellular damage but no evidence that PFOA increases the risk of clinically diagnosed liver disease	[54]
Nephrotoxicity	Odense Child Cohort (adults, *n* = 1612)	PFOA, PFOS	negative association between PFASs exposure (except for PFOA and PFDA) and estimated glomerular filtration rate (eGFR) and positive association with chronic kidney disease (CKD)	[55]
Asthma	Cross-sectional (children, *n* = 456)	PFOS, PFOA, PFBS, PFDA, PFNA, PFHxS and other	significant inverse association between serum PFASs and CC16 (club cell secretory protein; biomarker of asthma) levels in asthmatics	[56]
	Cross-sectional (children, *n* = 300)	PFOA, PFOS	positive association between serum PFASs level and impaired lung function in children (association was significant only in asthmatic children)	[57]
Immunotoxicity	Cross-sectional (adult, *n* = 733)	PFOA, PFOS, PFHxS, PFNA, PFDA and other	strong positive associations between blood PFOS level and leucocyte telomere length	[58]
	Odense Child Cohort(mother–child, *n* = 349)	PFOS, PFHxS, PFOA, PFNA, PFDA	deficient antibody responses in children prenatally exposed to PFASs	[59]

PFUnA, perfluoroundecanoic acid; PFDeA, perfluorodecanoic acid.

**Table 3:** Chemical and physical properties of compounds that belong to the PFASs group, most frequently determined in the human blood serum.

	PFOA	PFOS	PFBS
**Chemical Properties**			
Chemical Abstracts Services Number (CAS. No.)	335-7-1	2795-39-3	375-73-5
Physical state (at 20–25 °C)	white powder	white powder	liquid
Molecular weight (g/mol)	414	538	338
Solubility in water (at 25 °C; g/L)	9.5	0.550–0.570	Fully miscible
**Physical Properties**			
Melting point (°C)	45–54	>400	−21
Boiling point (°C)	188–192	not measurable	not measurable
Organic-carbon partition coefficient (log Koc)	2.06	2.57	2.7–3.6
Biochemical half-life	water: >92 years (at 25 °C) atmospheric: 90 days	water: >41 years (at 25 °C) atmospheric: 114 days	water:> 1 year (at 25 °C) atmospheric: 76.4 days

**Table 4:** Mean PFASs elimination half-lives (years or days). * Regards high exposure, based on Reference [108].

Elimination (t_1/2_, Days)	PFOA	PFHxA	PFBA	PFOS	PFHxS	PFBS
Human	2.1–3.9 y	14–49 d *	3–4 d	3.3–27 y	7.7–15.5 y	26 d
Monkey	21 d	1 d	2 d	45 d	100–141 d	4 d
Rat	5 d	0.2–0.05 d	0.3 d	24–82 d	0.9–34 d	0.02–0.3 d

**Table 5:** Occurrence of selected PFASs in human serum and urine.

PFASs	Place of Study	Year of Samples Collection	Level	Reference
Serum				
PFOA	New York, USA (occupational exposure)	2000–2002	8.1 ng/L	[135]
	USA (children serum, 3–11 year)	2013–2014	1.92 ng/mL	[136]
	New Hampshire, USA	2015	3.09 μg/L	[137]
	Slovakia (cord study)	2010–2012	0.79 ng/mL	[138]
	Australia (cohort study)	2014–2015	2.03 ng/mL	[139]
PFOS	New York, USA (occupational exposure)	2000–2002	34.3 ng/L	[135]
	Taipei, Taiwan	2009–2010	28.9 ng/mL	[140]
	Spain (cohort study)	2009–2010	7.61 ng/mL	[141]
	USA (children 3–11 year)	2013–2014	3.88 ng/mL	[136]
	New Hampshire, USA	2015	8.59 μg/L	[137]
	Slovakia (cord blood)	2010–2012	0.36 ng/mL	[138]
	Australia (cohort study)	2014–2015	5.24 ng/mL	[139]
PFBS	Serum (Taipei, Taiwan)	2009–2010	0.5 ng/mL	[140]
	Taipei, Taiwan	2009–2010	1.3 ng/mL	[140]
	USA (children serum, 3–11 year)	2013–2014	0.843 ng/mL	[136]
	Spain (cohort study)	2009–2010	0.836 ng/mL	[141]
	Australia (cohort study)	2014–2015	2.05 ng/mL	[139]
	Slovakia (cord blood)	2010–2012	0.07 ng/mL	[138]
PFNA	Taipei, Taiwan	2009–2010	0.8 ng/mL	[140]
	USA (children 3–11 year)	2013–2014	0.794 ng/mL	[136]
	Spain (cohort study)	2009–2010	0.954 ng/mL	[141]
	Slovakia (cord blood)	2010–2012	0.20 ng/mL	[138]
	Australia (cohort study)	2014–2015	0.49 ng/mL	[139]
Urine				
PFOA	China	2015	4.61 ng/L	[142]
	China	2011	12.9 ng/L	[143]
	Decatur, USA	2016	0.027 µg/L	[106]
PFOS	China	2015	18.71 ng/L	[142]
	China	2011	49.6 ng/L	[143]
PFHxS	China	2015	<1.41 ng/L	[142]
PFNA	China	2015	0.46 ng/L	[142]

**Table 6:** Different concentrations of PFASs determined in human serum from blood of mothers and umbilical cord blood. BQL—below method quantitation limit; *—median of measured concentration.

PFASs	Maternal Serum Mean (ng/mL) (max/min)	Cord Serum Mean (ng/mL) (max/min)	References
PFOA	1.22 (1.045/7.31)	0.919 (0.311/7.06)	[199]
	1.560 (1.045/7.31)	1.237 (0.237/2.878)	[193]
	2.8 (1.2/6.7)	3.10 *	[200,201]
	4.80 *	0.6/10.56	[200,202]
PFOS	3.67 (3.064/24.5)	1.28 (0/8.04)	[199]
	8.670 (1.728/22.857)	3.668 (0.535/12.674)	[193]
	12.70 *	3.5 *	[200]
	3.35	0.53/4.71	[202]
PFHxS	2.28 (0.619/31)	1.19 (0/16)	[199]
	0.528 (BQL/1.149)	0.331 (BQL/1.070)	[193]
	1.20 *	0.60 *	[200]
	2.24	0.05–1.93	[202]
PFNA	0.519 (0.430/3.29)	0.266 (0/2.25)	[199]
	0.41 (0.08/1.4)	0.41 *	[199]

## Abbreviations

AMPA	α-amino-3-hydroxy-5-methyl-4-isoxazolepropionic acid
AR	androgen receptor
BMI	body mass index
BPA	bisphenol A
BPAF	bisphenol AF
DHEA	dehydroepiandrosterone
DHEAS	dehydroepiandrosterone-sulfate
DHT	5α-dihydrotestosterone
EDCs	endocrine disrupting chemicals
ER	estrogen receptors
Erα	estrogen receptor α
Erβ	estrogen receptor β
FSH	follicle stimulating hormone
FT4	free thyroxine
FXR	farnesoid X receptor
GluR2	glutamate receptor 2
HDL	high-density lipoprotein
IARC	International Agency for Research on Cancer
LH	luteinizing hormone
LPL	lipoprotein lipase
LTP	long-term potentiation
LXR	liver X receptor
NMDAR	N-methyl-d-aspartate receptor
PBMCs	peripheral blood mononuclear cells
PFAA	perfluoroalkyl acids
PFASs	per- and polyfluoroalkyl substances
PFBS	perfluorobutane sulfonic acid
PFCAs	perfluoroalkyl carboxylic acids
PFDA	perfluorodecanoic acid
PFHxA	perfluorohexanoic acid
PFHxS	perfluorohexane sulfonic acid
PFOA	perfluorooctanoic acid
PFOS	perfluorooctanesulfonic acid
PFOSA	perfluoroalkane sulfonamide acids
PFSAs	perfluoroalkane sulfonic acids
POPs	persistent organic pollutants
PPARα	proliferator-activated receptor alfa
PPARγ	proliferator-activated receptor gamma
ROS	reactive oxygen species
RXR	retinoid x receptor
T3	free triiodothyronine
T4	total thyroxine
TDI	tolerable daily intake
THs	thyroid hormones
TR	thyroid hormone receptor
TSH	thyroid stimulating hormone
TTE	transplacental transfer efficiency
TWI	tolerated weekly intake
8-OHdG	8-hydroxy-2’-deoxyguanozine
